# Effect of Anthropometric Parameters on Achilles Tendon Stiffness of Professional Athletes Measured by Shear Wave Elastography

**DOI:** 10.3390/jcm12082963

**Published:** 2023-04-19

**Authors:** Claudia Römer, Enrico Zessin, Julia Czupajllo, Thomas Fischer, Bernd Wolfarth, Markus Herbert Lerchbaumer

**Affiliations:** 1 Department of Sports Medicine, Charité Universitätsmedizin Berlin, 10115 Berlin, Germany; 2 Department of Radiology, Charité–Universitätsmedizin Berlin, Charitéplatz 1, 10117 Berlin, Germany

**Keywords:** elastography, shear wave elastography, SWE, tendon, ultrasound, professional athlete

## Abstract

Background: Shear wave elastography (SWE) is currently used to detect tissue pathologies and, in the setting of preventive medicine, may have the potential to reveal structural changes before they lead to functional impairment. Hence, it would be desirable to determine the sensitivity of SWE and to investigate how Achilles tendon stiffness is affected by anthropometric variables and sport-specific locomotion. Methods: To investigate the influence of anthropometric parameters on Achilles tendon stiffness using SWE and examine different types of sports to develop approaches in preventive medicine for professional athletes, standardized SWE of Achilles tendon stiffness was performed in 65 healthy professional athletes (33 female, 32 male) in the longitudinal plane and relaxed tendon position. Descriptive analysis and linear regression were performed. Furthermore, subgroup analysis was performed for different sports (soccer, handball, sprint, volleyball, hammer throw). Results: In the total study population (n = 65), Achilles tendon stiffness was significantly higher in male professional athletes (*p* < 0.001) than in female professional athletes (10.98 m/s (10.15–11.65) vs. 12.19 m/s (11.25–14.74)). Multiple linear regression for AT stiffness did not reveal a significant impact of age or body mass index (BMI) (*p* > 0.05). Subgroup analysis for type of sport showed the highest AT stiffness values in sprinters (14.02 m/s (13.50–14.63)). Conclusion: There are significant gender differences in AT stiffness across different types of professional athletes. The highest AT stiffness values were found in sprinters, which needs to be considered when diagnosing tendon pathologies. Future studies are needed to investigate the benefit of pre- and post-season musculoskeletal SWE examinations of professional athletes and a possible benefit of rehabilitation or preventive medicine.

## 1. Introduction

The Achilles tendon (AT) is the largest tendon in the body, making it very easily accessible and suitable for ultrasound (US) examinations. Achilles tendinopathy is very common in both active individuals and in the general population and causes pain and severe limitations in mobility. Acute and chronic Achilles tendinopathy is one of the most common overuse injuries in professional athletes such as runners, jumpers and triathletes [1]. Runners have a lifetime prevalence of developing Achilles tendinopathy of 52% [2,3]. Furthermore, AT rupture is one of the most severe injuries of the lower limb, in terms of loss of training days in triathletes [4].

Tendon disorders cause pain and severe limitations in mobility. Since the pathogenesis of tendinopathy is considered a multifactorial process with inflammation and degeneration [1,5,6], established ultrasound techniques and MRI are limited tools in assessing morphological changes, which are required for clinical diagnosis of tendinopathies [7,8]. As it is reported that there are discrete areas of pathology in disordered tendons, it is even more relevant that SWE can provide direct measurements of specific areas within the tendon [2]. Shear wave elastography (SWE) has been reported to be a suitable technique to assess tissue stiffness and to allow the identification of injuries in professional athletes [9,10]. SWE has shown its potential and is already established in breast, liver, thyroid and prostate imaging [7]. Dirrichs et al. showed that SWE had higher sensitivity than B-mode and power Doppler US, which were established for the examination of tendinopathy [10]. In contrast to strain elastography (also known as compression elastography), in which the tissue is subjected to strain stress with uniform repetitive pressure and the resulting compression/deformation can be recorded, SWE uses a different basic physical principle. In addition to the ultrasound wave, another low-frequency shear wave is applied; the modulus of elasticity can be calculated from the propagation speed of this transverse wave [11]. Formed by ultrasonic pulses radiated perpendicular to the surface, the propagation of the shear wave front can be recorded and its propagation velocity calculated, which correlates with the modulus of elasticity. The technical advantage of the method is the independence of the externally applied pressure and thus an improved standardization with less susceptibility to inter- and intra-observer variability.

SWE has revealed significantly lower tendon stiffness in individuals with symptomatic Achilles lesions [10]. While the effects of anthropometric parameters such as age or body mass index (BMI) on tissue stiffness assessed by SWE have been investigated in several studies [12,13,14,15], data for professional athletes are missing. Softening of tendon tissue has been attributed to very early changes in tissue elasticity in early tendinopathy [5,16]. Tendons and muscles undergo changes in composition and architecture with aging, which impacts their mechanical properties and function [12,17]. Muscle mass declines with age, which leads to a progressive reduction of muscle function and strength. These changes impair daily life in the elderly. On the other hand, it has been shown that tendons maintain their dimensions and mechanical properties with aging [15,18]. Mechanical stress such as regular exercising can modulate age-related alterations and counteract a loss of function of the muscle–tendon unit [12]. In another study, participants with Achilles tendinopathy were older and had significantly higher BMIs compared to a control group [2]. The body fat percentage seemed to be more relevant for tendon stiffness than BMI, which could be explained by metabolic pro-inflammatory effects due to larger amounts of adipose tissue. 

Shear wave elastography holds great potential for detecting early changes in tendon structure, even before functional impairment becomes apparent [9].

Due to the increasing availability of SWE in commercial US systems, the number of publications on the topic of elastography increased in recent years. Despite this, musculoskeletal imaging (MSK) has seen only a limited increase in the level of evidence in a small number of musculoskeletal questions [19]. However, with the increasing use of elastography in musculoskeletal US, the growing number of studies may have the potential to establish this application in routine clinical practice for diagnosis and prevention. 

Before SWE can be used to diagnose soft tissue injuries such as tendinopathy, we need to develop valid diagnostic criteria to differentiate between healthy and abnormal tendons and to identify preclinical changes. To establish such criteria, we need to know how anthropometric parameters alter tissue stiffness in professional athletes and in different types of sports.

### Objective

The objective of this study is to investigate the influence of anthropometric parameters on the tendon and muscle stiffness of the lower limb using SWE and to determine the reference. Standardized SWE examinations of Achilles tendons in the longitudinal plane and relaxed tendon position were performed in 65 healthy professional athletes. 

## 2. Methods

### 2.1. Study Population

The prospective study included 65 healthy professional athletes, who were examined at Charité–Universitätsmedizin Berlin. Inclusion criteria were: (I) healthy professional athletes, (II) without any acute (>6 months) musculoskeletal, rheumatic or vascular comorbidities and no previous injuries of the Achilles tendon and (III) written informed consent to participate in the study. Exclusion criteria were: tendon neovascularization, hypoechogenity and tendon thickening. The study was conducted in accordance with the Declaration of Helsinki and was approved by the local ethics committee of Charité University Medicine Berlin (ethical vote number EA2/162/19).

Baseline participant characteristics were obtained by a questionnaire at the time of examination. On the day of the examination, no training was performed. Professional athletes (handball, soccer, volleyball, sprint, hammer throw) with more than 10 h training per week were included. All measurements were jointly performed by a trained sonographer and a highly experienced radiologist who were blinded to the type of sport.

### 2.2. Shear Wave Elastography Examination

All US-SWE examinations were performed using a standardized protocol. For assessment of the Achilles tendon (mid portion), participants were examined in the prone position with both feet hanging over the edge of the examination couch in a relaxed position. Prior to US-SWE, gray-scale B-mode US was performed in the transverse and longitudinal planes for the adequate assessment of the Achilles tendon and probe position. All examinations were performed using a high-end US system with a 4–10 MHz multifrequency linear array transducer and a center frequency of 7 MHz (Acuson Sequoia, Siemens Healthineers, Erlangen, Germany). The US-SWE software (Virtual Touch™) allows real-time measurement using Acoustic Radiation Force Impulse (ARFI) imaging technology for the quantitative evaluation of shear wave speed.

**Figure 1:**
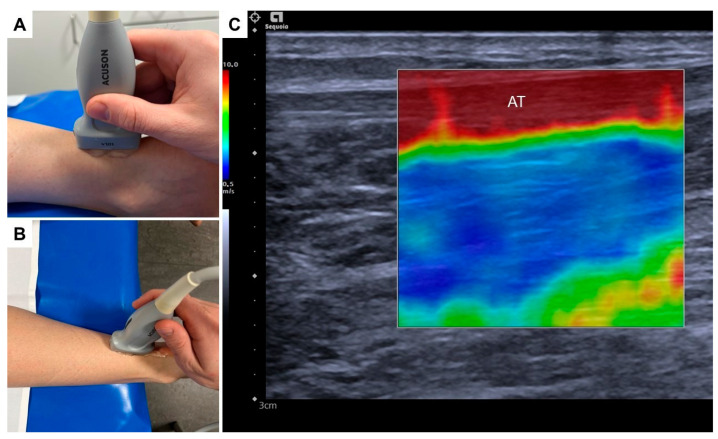
Example of SWE examination of a female athlete in a relaxed prone position with longitudinal probe orientation (**A**,**B**) and corresponding color-coded mapping of SWE (**C**) of the Achilles tendon (AT) in the mid-portion.

US-SWE examinations were performed in the longitudinal plane to depict each tendon and the area of interest in one single image (Figure 1). Using the respective 2D SWE approach, the examiner acquired four US images of the AT of the right leg of each professional athlete, with a total of 650 consecutive SWE measurements using a 3-mm circular region of interest (ROI) placed in the center of each target tendon, avoiding areas of visible artifacts. Thus, representative tendon stiffness is given as the median of 10 measurements and the corresponding interquartile range (IQR). Before ROI placement, shear wave speed as a surrogate for tissue stiffness was depicted by color-coded SWE mapping. The standardized penetration depth was adapted to each participant for optimal visualization of the tendon and correct SWE measurement. Gain was not changed to avoid potential effects on US-SWE measurement.

### 2.3. Statistical Analysis

Multiple linear regression analysis of AT stiffness was performed using anthropometric parameters such as gender, age and BMI as input parameters. Continuous variables were tested for normal distribution using the Kolmogorov–Smirnov test. Non-normally distributed variables are reported as median and IQR. A two-sided significance level of α = 0.05 was determined as appropriate to indicate statistical significance. All statistical analyses were performed using the SPSS software (IBM Corp., released 2019. IBM SPSS Statistics for Windows, Version 26.0. Armonk, NY: IBM Corp.) and Matlab (MATLAB and Statistics Toolbox Release 2022b, The MathWorks, Inc., Natick, Massachusetts, United States).

## 3. Results

### 3.1. Athletes’ Characteristics

A total of 65 professional athletes with a mean age of 20.19 years [16,17,18,19,20,21,22,23,24,25,26,27,28,29] were examined. The median BMI was 22.85 kg/m^2^ (IQR 19.60–32.38 kg/m^2^). The results are summarized in Table 1. One professional athlete had a history of hypothyroidism, while no other diseases such as diabetes mellitus, fatigue, hyperlipidemia, rheumatic diseases or malposition of lower limb joints were known. Overall, 14 athletes reported rupture of lower limb ligaments (ligament rupture of the ankle [n = 8), knee ligament rupture [n = 7]). None reported Achilles tendon pain, swelling, difficulty in joint movement or tendon rupture. Medications taken at the time of the examination were: hormonal contraceptives (oral [n = 9], intrauterine device [n = 2]).

### 3.2. Results of US-SWE in Professional Athletes

The analysis of variance (ANOVA) for AT stiffness in professional athletes is shown in Table 2 and Table 3. In multiple linear regression for AT stiffness, only gender showed a significant influence (*p* < 0.01), while age, height, weight and BMI did not (*p* > 0.05). Therefore, subgroup analysis for type of sport was performed for female and male professional athletes.

**Figure 2:**
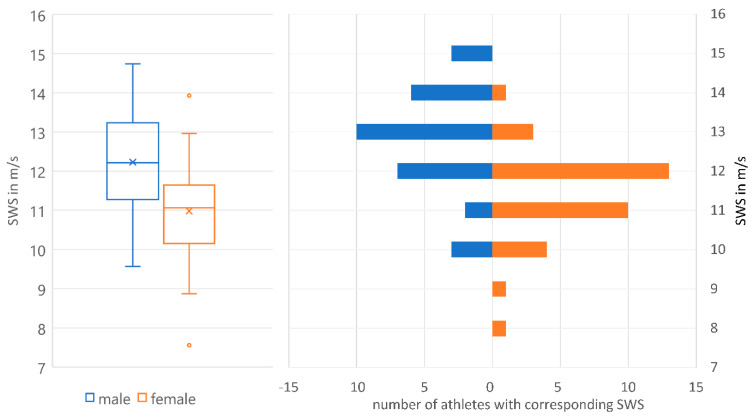
SWS in m/s of Achilles tendon in professional athletes (male = blue, female = orange).

Shear wave speed (SWS) for male and female athletes showed normal distribution (Figure 2). Male athletes had significantly higher AT SWE values, which can be seen in the boxplot in Figure 2. There was no significant difference in age and BMI between males and females (*p* > 0.05).

**Figure 3:**
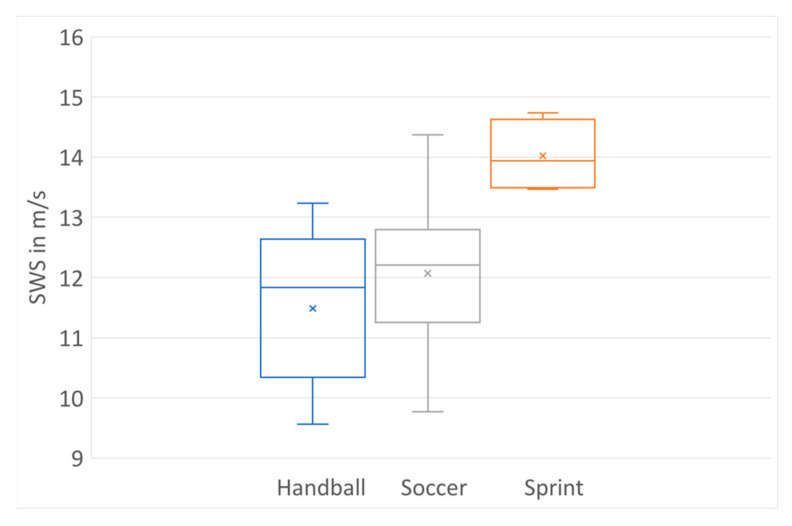
AT stiffness for male athletes in different sports.

For different types of sports and different load impacts on ATs, subgroup analysis was performed. In the male group, (sports: football (n = 21), handball (n = 7) and sprint (n = 4)) professional sprint athletes (14.02 m/s (13.50–14.63) showed significantly higher AT stiffness compared with handball (11.49 m/s (10.34–12.64), *p* < 0.01) and football players (12.06 m/s (11.25–12.87), *p* < 0.01), shown in Figure 3. 

**Figure 4:**
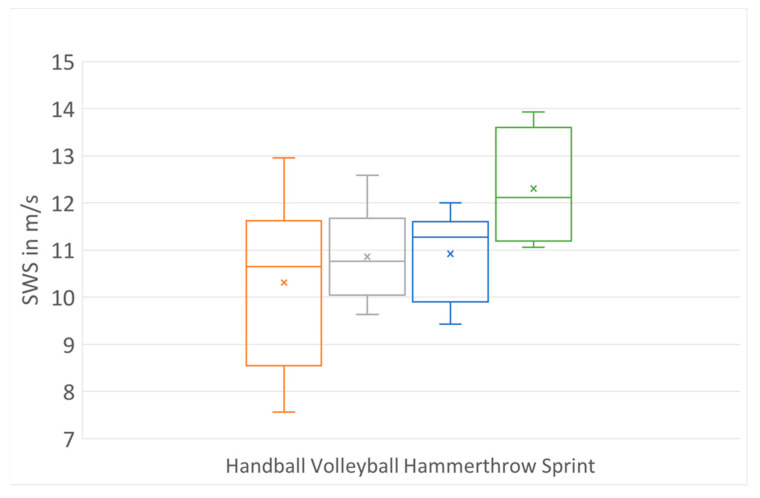
AT stiffness values for female athletes in different sports.

**Figure 5:**
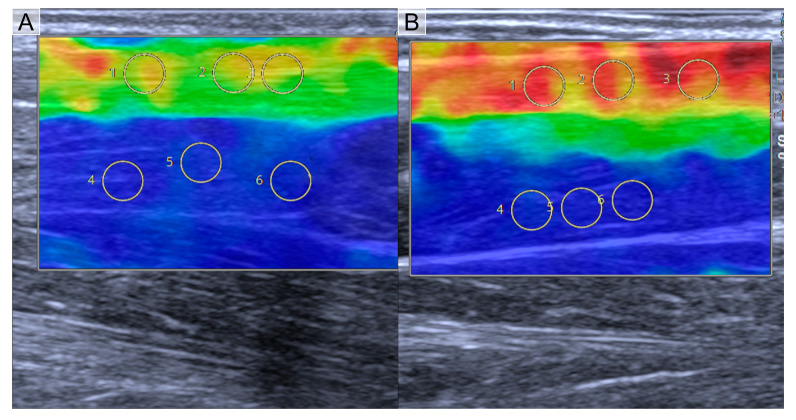
Color-coded mapping shows lower stiffness in SWE assessment in a female handball player (**A**, median SWS 10.31 m/s visualized as mostly green) and a female sprinter (**B**, median SWS 12.31 m/s visualized as mostly orange/red).

In the female group (sports: handball (n = 6), volleyball (n = 14), sprint (n = 4) and hammer throw (n = 9)), professional sprint athletes (12.31 m/s (11.19–13.60)) showed significantly higher AT stiffness compared with volleyball and handball players (VB: 10.88 m/s (10.04–11.68), HB: 10.31 m/s (8.57–11.63), *p* < 0.05) and hammer throwers (11.03 m/s (10.30–11.61), *p* < 0.05), (Figure 4 and Figure 5). No significant difference in AT stiffness was found between volleyball players and hammer throwers, volleyball and handball players and handball players and hammer throwers (*p* > 0.05).

## 4. Discussion

The role of SWE imaging in musculoskeletal applications is currently under discussion. In clinical practice, it is widely used for the assessment of Achilles tendinopathy and as an additional tool to confirm findings of B-mode power Doppler US of tendons. Available data were typically obtained in smaller study populations, and there is no guideline on musculoskeletal elastography in general for a standardized clinical application. Before we can use tendon stiffness measured by SWE in rehabilitation or injury prevention, we need to establish baseline values and determine the effects of demographic characteristics such as age, sex or BMI for professional athletes. 

### 4.1. Influence of Anthropometric Parameters on Achilles Tendon Stiffness

Our results show a significant effect of gender on AT stiffness. Stiffness values in male professional athletes were significantly higher than in female professional athletes (*p* < 0.001). This result is in line with the tendon stiffness reported for a non-athlete study population of similar size [20,21]; however, not in all degrees of dorsiflexion [20]. In another study of a non-athlete population, no significant difference in tendon stiffness was found between men and women [22]. A further study reported significant stiffness differences between professional and semi-professional athletes due to training load [23]. Training intensity is a relevant factor for Achilles tendon morphology [24], which is why caution is in order when comparing results obtained in semi-professional and professional athletes. Tendons combine elastic and viscous characteristics when undergoing deformation due to stress, so-called viscoelasticity. They transfer forces generated by muscles to bones to perform movements. The tendon structure is characterized by parallel bundles of collagen (30%) and elastin (2%), which are embedded in an extracellular matrix (68%) (water, tenocytes, mucopolysaccharide, proteoglycan gel). Mechanic loading is essential to maintaining tendon homeostasis [25]. Changes in this molecular structure have an impact on tendon stiffness and function [15]. 

Lower stiffness of the Achilles tendon has been reported for older individuals [12]. In our study, ANOVA did not identify age as a relevant parameter, which may be attributable to the fact that we investigated a significantly younger study population (16–29 years), which is significantly younger than other studies (20–85 years) [12,13,14]. In a study of 326 healthy volunteers, Fu et al. found no correlation between shear wave velocity and age [22]. Passive tissue stiffness and the correct measurement angle especially need to be considered [14] to assess further examinations in older professional athletes. Furthermore, no significant influence of BMI on tendon stiffness was found, as earlier studies also concluded [24,26,27]. Regular pre- and post-season preventive SWE examinations of the sport-specific exposed tendons in professional athletes may reduce injury risk by detecting early changes in tissue stiffness [9,28].

### 4.2. Achilles Tendon Stiffness in Different Sports

Tendon thickness and stiffness correlate positively with the strength of the corresponding muscle and might affect muscle function and force output, especially in the early phases of muscle contraction [25,29]. Besides gender, training load and maturation have different effects on the physical, chemical and mechanical properties of musculoskeletal tissue [24,30]. The elastic properties of tendons and muscles are influenced by activity level and show higher tissue stiffness in athletes [23,31]. Tissue stiffness might also be modulated by other factors including false locomotion patterns and sport types with high vertical forces, such as sprinting [32,33]. Athletes with greater mechanical stress and repetitive microtraumas show tendon thickening as part of structural remodeling processes and an increase in cross-sectional area [34]. Regular strength training and loading of muscles and tendons is known to lead to increased tendon size [18]. This increase is considered a compensatory mechanism to reduce mechanical loads on tendons and deformation resulting from increased body mass or muscle strength [29,35]. Therefore, we performed subgroup analysis for sports type to investigate AT stiffness in relation to different locomotion patterns, as all athletes included had training loads of >10 h per week in our study. AT stiffness values of professional female sprinters were significantly higher than in other sports such as handball, volleyball and hammer throw. The significant difference we found between professional sprinters and professional male soccer players is remarkable, as training patterns and running workload are comparable in these sports [36]. However, the running surface seems to have an important impact on AT maturation, stiffness and injury [33,37] and variable sprinting patterns from different angles, especially in a soccer game, needed to be considered and may influence tendon load and stiffness [36]. Soccer involves more stop-and-go movement, which may be a higher risk for injury [38] and could explain the lower AT stiffness values. Achilles tendon rupture is a critical injury for athletes, with return-to-sport rates of around 70% [39]. Return-to-sport is not exactly defined considering return-to-play in the same sports and at the same intensity as before [38]. In this context, AT tendinopathy and rupture can end an athlete’s career [39]. Two studies reported the strength of the lower limb with AT rupture to be significantly lower in comparison to the other limb after conservative or surgical therapy [38,40]. Sport-specific return-to-play guidelines are necessary to ensure optimal rehabilitation for injured athletes [39], and objective criteria are needed [41,42]. With both surgical and conservative treatment, AT rehabilitation is a complex and long process [43,44,45]. SWE can be a useful tool to regularly monitor tendon stiffness changes during rehabilitation, as stiffness might be the best parameter to assess the multifactorial risk factors for AT injuries [33]. Further studies are necessary to investigate the potential of SWE in rehabilitation after acute or chronic tendinopathy and in the rehabilitation process after AT rupture in professional athletes.

Not only do stiffness differences between professional and semiprofessional athletes need to be considered when SWE is used to detect abnormalities [10], but also differences in stiffness between different sports. Overall, SWE is an easy-to-use US technique to assess tissue stiffness in rehabilitation and preventive medicine. It is characterized by high intra- and inter-operator reliability [46], allowing faster and more cost-efficient diagnosis than MRI. 

### 4.3. Role of SWE in Assessment of Achilles Tendon

US has been considered the primary imaging modality of choice, with improved diagnostic performance in the evaluation of tendinopathic changes. Furthermore, the dynamic assessment established US as part of the functional investigation in acute ruptures linked to clinical examination as a point-of-care US tool. Compared to other anatomical regions such as the patellar tendon or quadriceps tendon, there are a larger number of studies for AT, resulting in a higher experience in Achilles tendon diagnostics, especially for tendinopathies. The healthy AT usually provides a more homogeneous color map with higher stiffness values compared to the patellar tendon or quadriceps tendon, whereas the stiffness of the tendon is related to the training volume or a relevant preload and can change, especially due to intensive training [23]. Thus, in professional athletes with a high training volume, the baseline stiffness is higher than in semi-professional amateur athletes. 

The use of SWE achieves high specificity in detecting tendinopathy, and the combined use of conventional US is recommended to increase the sensitivity of the diagnosis. In a meta-analysis, almost all studies described significantly reduced stiffness values in symptomatic tendons in the setting of tendinopathy, which also reflects clinical experience [47]. SWE can easily be used for diagnostic and follow-up purposes to demonstrate early changes in affected tendons and/or adaptation to the healthy contralateral side in the context of short-term follow-up. 

Although acute Achilles tendon rupture is often unequivocal on B-mode imaging and dynamic examination, SWE can also be helpful in this setting. Total rupture usually shows very low SWE values due to complete retraction and loss of tendon tension. This can be used especially in partial ruptures to differentiate the ruptured and still-preserved tendon portion, with corresponding higher stiffness values. SWE can be used in patients with AT rupture to assess contralateral tendon stiffness and elasticity. Interestingly, Ivanac et al. demonstrated a lower elasticity (−23%) of contralateral ATs in patients with acute AT rupture compared to healthy individuals based on SWE findings [48]. This study’s results show a potential disorder or compensation of contralateral tendons after surgery. Hence, contralateral tendons may be exposed to higher force transmission after surgery in patients who suffered from acute rupture. This may lead to a higher vulnerability for future ruptures on the contralateral side.

### 4.4. Future Aspects in SWE

Despite a great interest in SWE imaging, the published literature is still sparse and strongly focused on cross-sectional studies with small patient numbers. Thus, the clinically established application of SWE is mainly anchored in diagnostic questions of tendinopathy. Here, SWE is usually used additively to the established B-Mode and Doppler Imaging criteria and increases the diagnostic power. This addition of multiple newer US applications is now increasingly presented as “multiparametric ultrasound”. 

Currently, there is no guideline for SWE in musculoskeletal tissue. Furthermore, the metric values given by US systems from different manufacturers are not directly comparable. There is still no guideline in the field of MSK imaging that summarizes a consensus on technical principles (e.g., measurement field size, region of interest or number of measurements per area). In addition to the diagnostic approach of SWE, longitudinal studies are currently lacking, especially in the field of sports medicine, e.g., in the context of muscle injuries or prognostics (a possible preventive approach of SWE). The scientifically relevant question in the coming years will be whether the early use of SWE could prevent acute injuries in a pre-damaged tendon or an overloaded muscle. Quantitative assessment of tissue stiffness and elasticity allows metric assessment of intrinsic tissue properties, which may be of particular value in tissue healing or diagnosing early-stage disease if no pathological findings can be depicted in B-Mode US or Doppler imaging. SWE may help in the prediction of impending tendon failure, which probably helps the clinician in making decisions for the early initiation of treatment [19].

### 4.5. Limitations

Nine female professional athletes in our study took an oral hormonal contraceptive and this constitutes future work. Oral contraceptives have an impact on the natural fluctuation of hormones [49,50,51]. However, the exact effect on tendon stiffness is still a topic under debate due to less high-quality studies and contradictive results [51,52,53]. Other factors such as low energy availability and psychological stress, which can lead to higher cortisol levels and may also affect tissue properties, need to be considered [53,54]. We did not consider the menstrual cycle phases of female athletes, which will be considered in future studies. Further limitations of our study are the small sample sizes for the sports-specific subgroup analysis and the relatively high scatter of measured values. This results from the exclusion criteria of tendinopathy symptoms (pain, swelling) and ultrasound findings (neovascularization, hypoechogenicity and tendon thickening). Further investigations should include a larger number of athletes in different sports.

## 5. Conclusions

Gender and type of sports need to be considered as influencing factors when assessing AT stiffness by SWE in professional athletes. Especially for professional athletes, easy access to diagnostic tools is necessary to detect the early stages of injuries and to develop preventive treatment algorithms to avoid severe tendon and muscle injuries. Further studies are necessary to investigate larger groups of professional athletes in different sports.

## Figures and Tables

**Table 1:** Baseline characteristics of professional athletes (n = 65).

	Male (n = 32)		Female (n = 33)	
	Mean & Range		Mean & Range	
AT	12.19	11.25–14.74	10.98	10.15–11.65
Age	19.83	18.00–21.00	20.76	18.00–24.50
BMI	23.52	22.40–24.43	23.34	21.30–24.38

**Table 2:** Multiple linear regression model of AT stiffness in professional athletes.

	Coefficient	Standard Error	*p* Value
Bias	4.24	25.48	0.8686
Gender (m = 1, f = 0)	−1.34	0.42	0.0021
Age	0.02	0.05	0.7060
Height	4.40	14.09	0.7561
Weight	-0.05	0.16	0.7545
BMI	0.16	0.53	0.7697

**Table 3:** Linear regression model of AT stiffness in professional athletes only for gender.

	Coefficient	Standard Error	*p* Value
Bias	10.98	0.22	<0.0001
Gender (m = 1, f = 0)	1.20	0.32	0.0003
R² (adjusted)	0.17		
Standard error	1.29 m/s		

## Data Availability

The data presented in this study are available on request from the corresponding author. The data are not publicly available due to data privacy regulations.

## Abbreviations

AT	Achilles tendon
BMI	Body mass index
MSK	Musculoskeletal
ROI	Region of interest
SWE	Shear wave elastography
SWS	Shear wave speed
US	Ultrasound
